# H_2_O_2_-PLA-(Alg)_2_Ca Hydrogel Enriched in Matrigel^®^ Promotes Diabetic Wound Healing

**DOI:** 10.3390/pharmaceutics15030857

**Published:** 2023-03-06

**Authors:** Alexandra Cătălina Bîrcă, Cristina Chircov, Adelina Gabriela Niculescu, Herman Hildegard, Cornel Baltă, Marcel Roșu, Bianca Mladin, Oana Gherasim, Dan Eduard Mihaiescu, Bogdan Ștefan Vasile, Alexandru Mihai Grumezescu, Ecaterina Andronescu, Anca Oana Hermenean

**Affiliations:** 1 Department of Science and Engineering of Oxide Materials and Nanomaterials, Politehnica University of Bucharest, 011061 Bucharest, Romania; 2 Research Institute of the University of Bucharest—ICUB, University of Bucharest, 050657 Bucharest, Romania; 3 “Aurel Ardelean” Institute of Life Sciences, “Vasile Goldis” Western University of Arad, 310025 Arad, Romania; 4 Lasers Department, National Institute for Lasers, Plasma and Radiation Physics, 409 Atomistilor Street, 077125 Magurele, Romania; 5 Department of Organic Chemistry, Politehnica University of Bucharest, 011061 Bucharest, Romania; 6 Academy of Romanian Scientists, Ilfov No. 3, 050044 Bucharest, Romania

**Keywords:** alginate-hydrogel-based dressing, oxygen-enriched polylactic acid microspheres, Matrigel, skin, wound healing

## Abstract

Hydrogel-based dressings exhibit suitable features for successful wound healing, including flexibility, high water-vapor permeability and moisture retention, and exudate absorption capacity. Moreover, enriching the hydrogel matrix with additional therapeutic components has the potential to generate synergistic results. Thus, the present study centered on diabetic wound healing using a Matrigel-enriched alginate hydrogel embedded with polylactic acid (PLA) microspheres containing hydrogen peroxide (H_2_O_2_). The synthesis and physicochemical characterization of the samples, performed to evidence their compositional and microstructural features, swelling, and oxygen-entrapping capacity, were reported. For investigating the three-fold goal of the designed dressings (i.e., releasing oxygen at the wound site and maintaining a moist environment for faster healing, ensuring the absorption of a significant amount of exudate, and providing biocompatibility), in vivo biological tests on wounds of diabetic mice were approached. Evaluating multiple aspects during the healing process, the obtained composite material proved its efficiency for wound dressing applications by accelerating wound healing and promoting angiogenesis in diabetic skin injuries.

## 1. Introduction

As hydrogels exhibit similar hydration with skin tissue and stimulate the epithelization process, they represent an attractive choice for treating wounds of any type [1,2,3]. Moreover, their biomimicking microstructure, composition-related loading efficiency of the therapeutic cargo, and circumstantial stimuli-responsive capacity provide indisputable characteristics for fabricating advanced topical formulations [4,5].

In particular, hydrogels as dressings could be a viable option to control the water permeability in injured skin [6]. This is an essential aspect that must be addressed because injured skin causes considerable losses of water and fluids, about 20 times higher than those caused by healthy skin. Regularly, the daily amount of water that is usually lost through healthy skin is ~250 gm^−2^/day at 35 °C, while when the skin is affected, it can lose up to 5000 gm^−2^/day, depending on the wound type. These processes must be considered when designing a wound dressing toward endowing it with high water-vapor permeability and the ability to absorb exudate fluid without causing other symptoms or conditions to patients. Thus, a wound dressing must act as an adherent protective membrane and simultaneously provide the conditions for optimal healing, with this balance being a challenge for research in the field [7,8,9,10,11,12].

The naturally originating alginates are leading representatives of polysaccharide-based hydrogels [13]. The reaction occurring during the formation of alginate-based hydrogels is either ionic cross-linking mediated by COO^-^ groups or chemical cross-linking between additional side chains. Alginates exhibit promising features for the fabrication of performance-enhanced wound dressings. Specifically, given the intrinsic hydrophilicity, alginate-based hydrogels can absorb significant amounts of exudate released by the wound. Moreover, the alginate exerts a hemostatic effect when in contact with bleeding wounds and stimulates angiogenesis while promoting cell proliferation and collagen production. It also decreases the concentration of proinflammatory molecules (reactive species) and down-regulates the secretion of proinflammatory cytokines in chronic wounds. Additionally, the flexibility and easy removal make it a comfortable alternative to conventional wound dressings. However, alginates present one main drawback, namely the lack of adhesivity, therefore requiring the use of a secondary dressing or combination with other polymers to improve the mechanical properties [14,15,16,17,18].

Polylactic acid (PLA) is a synthetic polyester recognized for good biocompatibility and characterized by thermoplasticity and suitable mechanical properties, including strength [19]. This material has attractive medical applications, reportedly being used in biodegradable sutures, scaffolds, and a wide range of drug delivery systems [20,21]. The primary polylactic acid monomeric block is Lactide, characterized by L-lactide or D-lactide type. The chirality type differentiates between the properties of the polymer, such as mechanical properties, semi-crystalline/amorphous characteristics, and biodegradability. For instance, it has been shown that D and mixed L/D forms have a higher degradability rate than the L form. Compared to other polymers, the chirality-based chemical capacity of polylactic acid (L, D, or L/D) leads to semi-permeable properties to water and oxygen, thus being more susceptible to biodegradation compared to other polymers used in the medical field [22,23,24]. Moreover, the degradation of PLA can be enhanced by enlarging the surface area ratio or by increasing the porosity of the polymer. This is due to the fact that the degradation process is active both outside the surface and within the core of the polymer when the material shows porosity [25,26,27,28].

Another polymer of extensive use in biomedicine is polyvinyl alcohol (PVA), which shows promising outcomes in the field owing to its favorable properties, such as biocompatibility, non-toxicity, easy film-forming ability, high hydrophilicity, and mechanical and chemical resistance [29,30]. Given its versatile features, PVA is currently one of the oldest and most used synthetic polymers in biomedical applications, such as wound dressings, contact lenses, artificial organs, and drug delivery systems [31,32]. However, when blended with other polymers, such as PLA, synergistic benefits of the PVA-based platforms have been evidenced [33,34,35].

Besides choosing the optimum wound dressing substrate materials, the composite can be further enriched with bioactive molecules that augment the restorative potential and provide extensive therapeutic effects. For instance, the significant role of oxygen in modulating the wound healing process is well known, whether acute or chronic. Specifically, proper wound oxygenation contributes to the formation of granulation tissues and enhances collagen production, and also activates tissue-repairing pathways by providing sufficient energy levels to support cellular events [35,36]. Moreover, the local incidence of bacterial infection is significantly reduced, contributing to accelerated healing. In addition, oxygen supply stimulates the angiogenesis in the wound sites and determines bacteriostatic effects, being an essential factor for the physiological wound healing process [37,38,39,40]. Thus, fabricating a dressing with the ability to provide oxygen to the wounded site represents an appealing strategy to boost the healing process.

One of the challenges in wound healing is vascularization, which can be sustained or induced by a cocktail of immunomodulatory molecules and growth factors that play a role in various steps of angiogenesis. Matrigel, a sterile and soluble extract from the basal membrane matrix derived from the Englebreth–Holm–Swarm tumor, represents a potential choice to overcome the limitations that arise during the wound healing process. What is very important is that Matrigel contains a mixture of structural proteins and proteoglycans, and angiogenic growth factors, thus leading to the support and generation of new blood vessels [41,42,43,44]. Therefore, its incorporation into the wound dressing will result in improved vascularization of the affected tissue.

Out of the wide range of wound types, the diabetic foot ulcer represents a critical issue in wound care management. It occurs in 20–30% of diabetic patients and culminates in most cases with amputation surgery [45,46,47,48]. In diabetic patients, the inadequate glycemic level significantly affects the wound healing process by the impossibility of closing the ulceration. However, controlling glycemic levels is a serious challenge. Still, if this activity is maintained correctly, the benefits will appear in the patient’s quality of life regarding diabetic disease and chronic wound healing [49,50,51,52,53,54]. In this context, creating advanced wound dressings for diabetic foot ulcers is of tremendous importance, being a current focus of the scientific community and resulting in the elaboration of new and promising therapeutic approaches [55,56,57,58].

Taking everything into account, this study aims to develop performant polymer-based dressings that serve as platforms to treat diabetic ulceration with the help of oxygen activity. The struggle to evolve in the way of developing new materials and methods for improving the healing process of diabetic wounds led to obtaining some dressings that fulfill the crucial needs to have conclusive results. In this respect, three hydrogel formulations were developed herein (i.e., alginate hydrogel—HG, alginate hydrogel loaded with H_2_O_2_-PLA microspheres—HG_OMs, and alginate hydrogel loaded with H_2_O_2_-PLA microspheres and Matrigel—HG_OMs_MG) and characterized from the physicochemical and biological points of view.

## 2. Materials and Methods

### 2.1. Materials

In order to obtain the alginate-based hydrogels, which additionally include PLA microspheres that encapsulate H_2_O_2_, the following materials were used: polylactic acid (PLA), polyvinyl alcohol (PVA), chloroform (CHCl_3_), hydrogen peroxide (H_2_O_2_), sodium alginate, calcium chloride (CaCl_2_), and Matrigel Matrix Growth Factor Reduced (code BD 356321). All reagents were purchased from Sigma–Aldrich (Merck Group, Darmstadt, Germany) and used as-received, without additional purification.

The same supplier provided most of the reagents used during the in vivo assays (otherwise, the provider was properly mentioned).

To use the developed dressings as an effective remedy for skin defects, H_2_O_2_ was embedded in PLA microspheres that were further loaded in a multifunctional hydrogel matrix that could control the oxygen release and prevent primary infection of the wound surface while accelerating the healing process.

### 2.2. Synthesis of H_2_O_2_-PLA Microspheres (OMs)

A 2% (*w/v*) PVA solution in distilled water at 80 °C under continuous stirring was first prepared. Then, the H_2_O_2_ solution and 2% PVA solution were mixed at room temperature (1:1 volume ratio) to obtain the hydrophilic part of the microemulsion preparation.

Secondly, a 50 mg/mL PLA/CHCl_3_ mixture, representing the hydrophobic part, was prepared.

Both solutions were mixed and subjected to ultrasound, according to the following parameters: amplitude—50%, time of action—3 min (successive 3 s ON/3 s OFF steps). The sonicated solution was washed and centrifuged for 15 min, under 6000 rpm, finally obtaining a precipitate (coded as OMs) for further use and physicochemical analyses.

### 2.3. Preparation of OMs-Loaded Alginate Hydrogels (HG_OMs and HG_OMs_MG)

All hydrogel formulations were prepared starting from a 5 mg/mL alginate solution. Blank alginate solution resulted in the formation of HG hydrogel after CaCl_2_-mediated cross-linking. Further, H_2_O_2_-PLA microspheres dispersed in water were mixed with the polysaccharide solution (1:7 volume ratio) to obtain the physically cross-linked HG_OMs hydrogel. Finally, the HG_OMs_MG hydrogel was obtained using a similar protocol, but 2 mg of Matrigel was added.

Hydrogels were obtained in duplicate, one part being lyophilized and investigated from a physicochemical point of view and another part being tested from a biological point of view.

### 2.4. Physicochemical Characterization

#### 2.4.1. Fourier Transform Infrared Spectroscopy (FTIR)

To investigate the compositional integrity of the prepared formulations, sequential infrared studies were performed. A small amount of each sample was analyzed using a ZnSe crystal of the Nicolet 6700 FT-IR spectrometer purchased from Thermo Fischer Scientific (Madison, WI, USA). Measurements were performed at room temperature, with 32 sample scans/sample, between 4000 and 1000 cm^−1^ at a resolution of 4 cm^−1^. Recording and processing the information thus acquired was possible by using the OmnicPicta program (version 8.2, Thermo Fischer Scientific, Madison, WI, USA).

#### 2.4.2. Scanning Electron Microscopy (SEM)

Relevant information on the surface morphology and porosity of samples was obtained by SEM analysis using an FEI Quanta Inspect F50 electron microscope from Thermo Fischer Scientific (Hilsboro, OR, USA). The investigations were carried out in a high vacuum, using the secondary electron mode at 20 and 30 keV acceleration voltages. To reduce electrical charging through analysis, samples were capped with a thin gold film.

#### 2.4.3. Swelling Rate

A biopsy punch was used to obtain cylindrical sections (5 mm diameter) from the dressings, which were immersed in 2 mL of simulated body fluid (SBF, prepared according to Kokubo’s protocol) at a temperature of 37 °C. Samples were weighed before and after different time points of immersion in SBF, and their swelling rate was estimated using the formula:

(1) $$Swelling\;ratio=\frac{Wt-Wi}{Wi}\times 100\%$$

where *Wi* and *Wt* are the samples’ mass before (initial) and after (time-point) SBF immersion.

#### 2.4.4. Degradation Rate

Weighed HG and HG_OMs samples were immersed in SBF for 24 and 48 h, then dried and weighed again. Mass changes in dried hydrogels were used to estimate the incipient degradation of materials using the formula:

(2) $$Degradation=\left(1-\frac{W0-Wt}{W0}\right)\times 100\%$$

where *W*0 and *Wt* represent the mass of each hydrogel dressing before immersion in SBF and after immersion/drying at different intervals.

#### 2.4.5. Hydrogen Peroxide Quantification

Regarding the qualitative and quantitative analysis of H_2_O_2_, an HR-MS (FT-ICR) protocol was developed using the well-known salicylic acid color reaction of Fe^III^ ions after Fe^II^ oxidation by H_2_O_2_ [59]. The Fe^III^(Salyc)_2_ complex was identified (327.9867, 329.9820, and 330.9854 amu peaks) using as a reference the molecular cluster prediction from a simulation tool for high-resolution molecular clusters (Data Analysis software tool of the SolariX system). In addition, the fine isotopic structure of the 330.9854 amu peak was used as confirmation for the qualitative analysis approach (330.9825 amu—^12^C_14_^1^H_10_^57^Fe^16^O_6_, 330.9845 amu—^12^C_13_^13^C^1^H_10_^56^Fe^16^O_6_, 330.9884 amu—^12^C_14_^1^H_9_^2^H^56^Fe^16^O_6_). For mass calibration purposes, a NaTFA solution was used in positive ESI ionization mode). After the sample preparation step, the sample and all calibration samples were analyzed using FT–ICR MS, using the direct infusion sample introduction device and positive ESI ionization mode. For the quantitative analysis approach, the 329.9820 amu mass peak was used, and calibration was performed using different H_2_O_2_ concentrations in the 35–150 mg/L range, where a R^2^ = 0.9905 was obtained using the y = 0.0015x + 0.5054 regression trendline. The average mass resolution of the 329.9820 amu mass peak was 1,063,690 (sd 0.431) FWHM.

All HR-MS analyses were performed by an FT–ICR MS with a 15 T superconducting magnet (solar X–XR, QqqFT–ICR, Bruker Daltonics) with electrospray ionization (ESI) device and direct infusion for sample introduction. For the positive ESI ionization experiments, the sample was introduced at a sample flow rate of 120 µL/h, a nebulizing gas pressure (N_2_) of 2.5 bar at 200 °C, and a flow rate of 1 L/min. The spectra were recorded over a mass range between 250 and 480 uam at a source voltage of 3700 V [60].

### 2.5. Biological Evaluation

#### 2.5.1. In Vivo Experimental Design

The present study followed the ethics criteria of the Animal Facility regulation and Ethical Research Committee at Vasile Goldis Western University of Arad, Romania.

Male CD1 mice (weight 25~30 g, 8 weeks of age) were intraperitoneally injected with streptozotocin (STZ, Santa Cruz Biotechnology, the Netherlands; 102 mg/kg body weight) in 50 mM citrate buffer (pH 4.5) to induce diabetes. Four days after injection, blood glucose levels were measured using an Accu-check Blood Glucose Meter (Roche Diagnostics, Indianapolis, IN, USA). A diabetic phenotype in the animals was confirmed with a blood glucose level of over 400 mg/dL.

Diabetic mice were anesthetized with intramuscular ketamine hydrochloride and xylazine injection, and the surgical area was shaved. Full-thickness dermal wounds with a diameter of 3 mm were removed at four places on each side of the back created using a 3 mm biopsy punch, and fixed with a silicone ring to avoid wound contraction.

**Figure 1:**
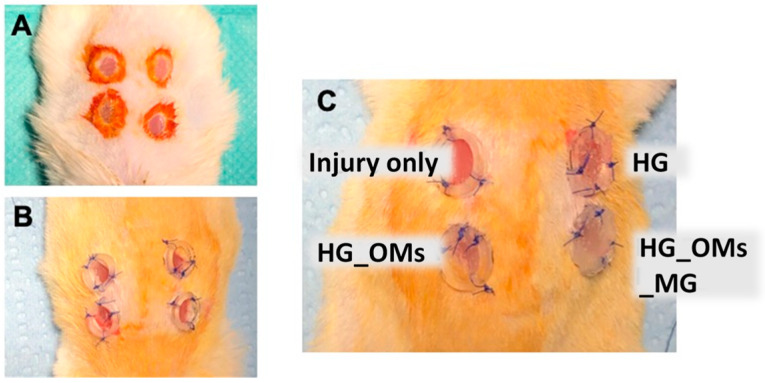
The surgical procedure used on the murine model. (**A**) Execution of the skin defect. (**B**) Placement of the silicone rings. (**C**) Placement of hydrogels.

Four types of skin wounds per animal were performed (Figure 1): 1. only the thick dermis was removed (control, C); 2. the wound was transplanted with alginate hydrogel (HG); 3. the wound was transplanted with oxygen-releasing alginate hydrogel, containing H_2_O_2_-PLA microspheres (HG_OMs); 4. the wound was transplanted with an oxygen-releasing hydrogel, containing H_2_O_2_-PLA microspheres and 4.44 µg/mg of Matrigel (HG_OMs_MG). The dressing covered just the area of the skin defect. After applying the primary dressing, the wound area was covered with gauze and secured by the adhesive bandage. On days 3 and 7, tissue samples were collected for histopathological analysis.

#### 2.5.2. Histopathology

The skin explants were washed with phosphate-buffered saline (PBS) and fixed in 4% paraformaldehyde (PFA) for 24 h. After fixation, samples were dehydrated after sequential immersion in increasing concentrations of alcohols, cleared, and further embedded in paraffin blocks. Histological sections (5 μm) were prepared using a microtome and subsequently stained with hematoxylin & eosin (H&E, BioOptica, Italy) for morphological analysis and Dane stain (Titolchimica, Italy, TC 20822) to highlight pre-keratin and keratin on wound sites. The differentiation of pre-keratin and keratin, obtained through the Orange G solution, is evidenced by color variations (orange and red-orange, respectively).

The stained sections were examined under light microscopy using an Olympus BX43 microscope (Olympus Life Science, Japan) and photographed using a digital camera (Olympus XC30).

## 3. Results

### 3.1. Physicochemical Characterization

**Figure 2:**
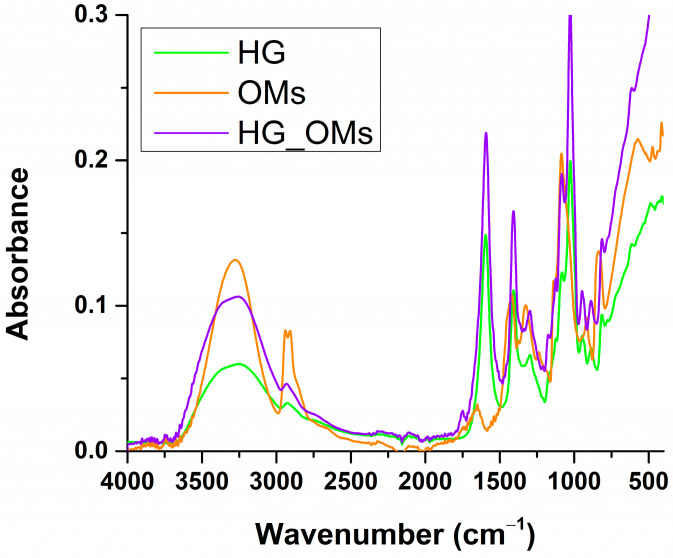
The FT-IR spectra of HG, Oms, and HG_OMs.

Figure 2 presents the FTIR spectra of OMs, HG, and HG_OMs samples. All spectra show a broad vibration band between 3100 and 3400 cm^−1^, characteristic of abundant OH groups originating from the hydrophilic polymers used in our study [61,62].

Vibrational bands between 1500 and 1750 cm^−1^ are characteristic of C=O groups specifically shown in the PLA structure (OMs sample) and COO^–^ moieties within the alginate (HG and HG_OMs samples). Overlapped infrared vibrations can be noticed at ~1400 cm^−1^ (symmetric deformation of PLA methyl and symmetric stretching of alginate-originating carboxyl) and between 1000 and 1100 cm^−1^ (C–O–C vibrations from both biopolymers and PVA-specific C–O stretching) [63,64]. The absorption bands in the 2800–3000 cm^−1^ wavenumber range are attributed to characteristic vibrations of the C–H groups.

**Figure 3:**
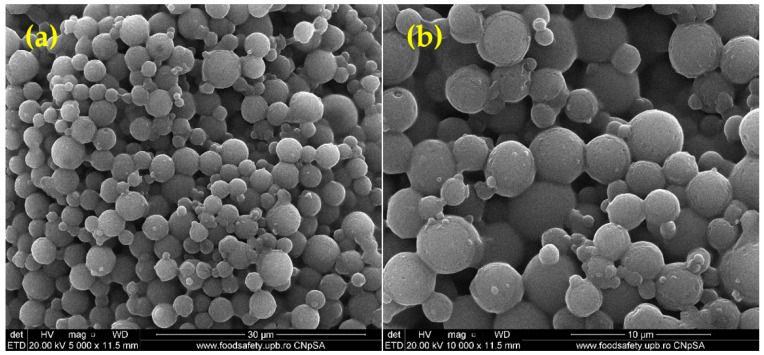
SEM images of OMs (**a**,**b**).

The SEM micrographs of PLA-based systems (Figure 3) show their spherical morphology and comparable dimensions. Polymeric spheres of micronic dimensions (1–3 µm), with smooth surfaces and no clear signs of structural damage, are obtained using an adapted emulsification protocol. At the same time, microsphere aggregates, consisting of interconnected particles, can be noticed.

**Figure 4:**
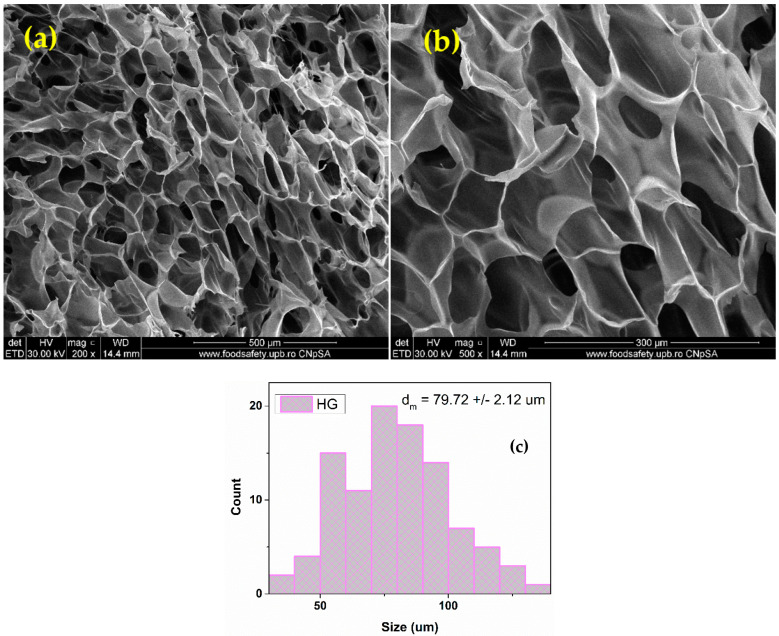
SEM images of HG control sample (**a**,**b**) and pore size distribution (**c**).

**Figure 5:**
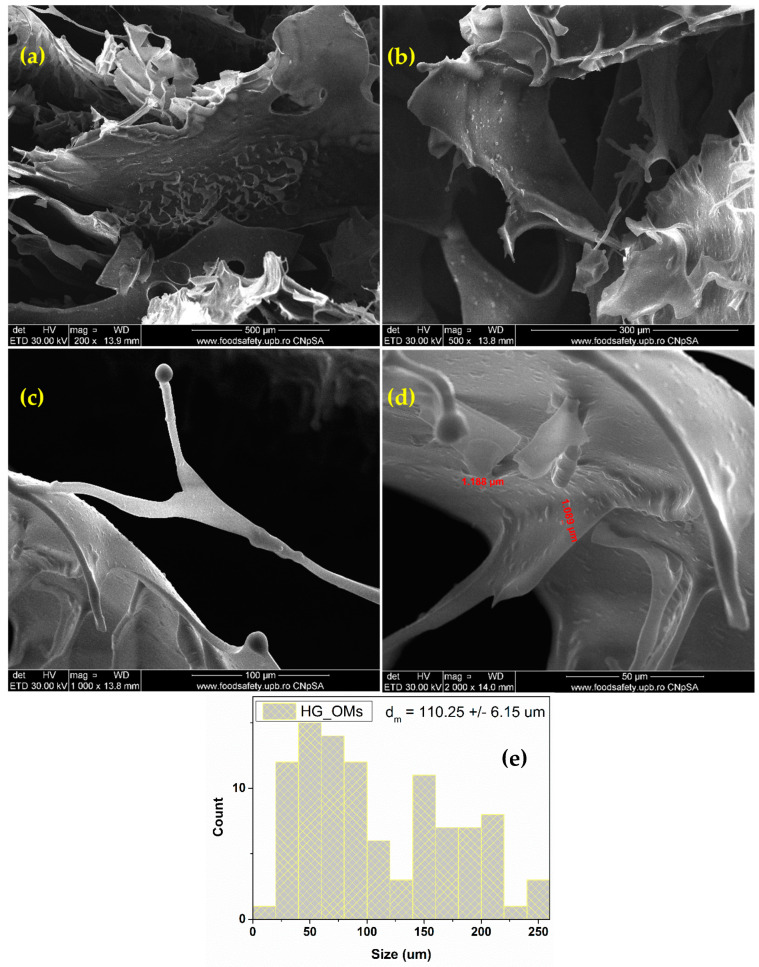
SEM images of HG_OMs (**a**–**d**) and pore size distribution (**e**).

The SEM analysis of the hydrogel dressings reveals that HG (Figure 4) and HG_OMs (Figure 5) samples show a highly porous structure, which is beneficial for stimulating the cell adhesion process and promoting cellular proliferation and migration. However, the main difference between these samples relies on the pore structure, which is significantly modified following the addition of PLA-based microspheres.

The collected micrographs show the uniform macroporosity and interconnected pore structure of the HG control sample (Figure 5), with a measured average pore size of 79.72 ± 2.12 µm. The smooth surface of pore walls can also be noticed.

As previously mentioned, a distinctive pore structure of alginate-based hydrogels is evidenced after the addition of OMs (Figure 5), resulting in an increased and more heterogenous porosity. Wider pores, having an average dimension of 110.25 ± 6.15 µm, have been estimated for the HG_OMs hydrogel. It can be seen that the spheres are evenly distributed on the surface of the hydrogel but also within the hydrogel matrix (evidenced by the presence of an irregular and textured surface of the pore walls). Correlated with the SEM data obtained for the hydrogel-free spheres, one can notice that the OMs spheres retain their micronic dimensions after loading in the hydrogel matrix, with values ranging from ~1 μm (spheres distributed within the hydrogel) to ~3 μm (spheres distributed on the surface of the hydrogel).

**Figure 6:**
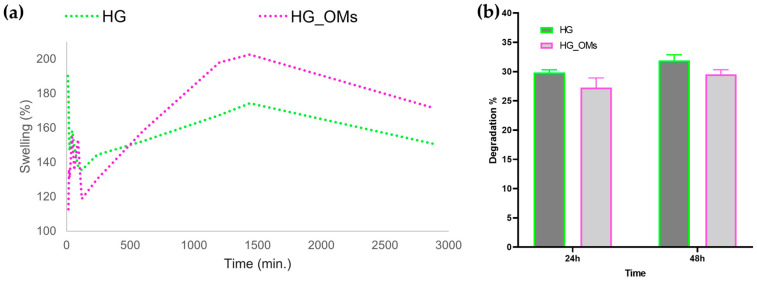
Swelling rate (**a**) and degradation (**b**) of HG and HG_OMs.

Mass variations of HG and HG_OMs soaked in SBF, directly correlated with the swelling rate in hydrogel formulations, are represented in Figure 6a. A time-dependent ability for progressive swelling is noticed for both samples, with a maximal swelling degree of ~140% (HG) and ~160% (HG_OMs) after 2 days. Complementary results evidence the time-dependent degradation of both hydrogels, with a reduced rate in the case of the HG_OMs dressing (Figure 6b).

### 3.2. Gross Morphological Analysis of the Wounds

**Figure 7:**
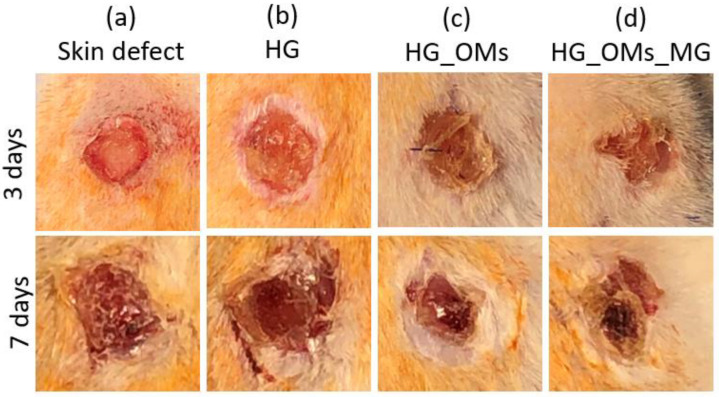
Gross morphology of wounds at different time points after surgery. (Right) Wound closures were observed on days 3 and 7 after the wound injury. (**a**) Full-thickness skin defect (control); (**b**) alginate hydrogel dressing (HG); (**c**) oxygen-releasing alginate hydrogel containing H_2_O_2_-PLA microspheres (HG_OMs); (**d**) oxygen-releasing hydrogel, containing H_2_O_2_-PLA microspheres and 4.44 μg/mg (HG_OMs_MG). (Left) Wound closure size (cm^2^).

Figure 7 shows the gross aspects of the hydrogel-dressing-treated wounds compared to full-thickness skin defects (control) at 3 and 7 days post-injury. The size of wounds treated with HG_OMs_MG is smaller than those treated with HG_OMs and HG, and the gross aspect of the skin is improved at both time intervals post-surgery. Moreover, the wound dressing with the alginate hydrogel containing oxygen-enriched microspheres and Matrigel is almost the same, the re-epithelization is near completion, and no edema, erythema, or other clinical signs of inflammation are observed.

### 3.3. Histological Aspect of the Wounds 3 Days after Injury (H&E Stain)

**Figure 8:**
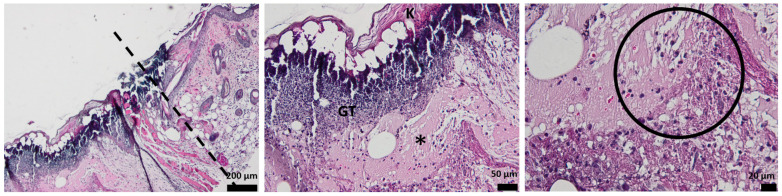
The histological aspect of the full-thickness skin defect at 3 days (H&E stain). Symbols: dotted line—separates healthy skin from the defect; GT—granulation tissue; *—edema; circle—neutrophils, K—keratin.

Histological analysis of the full-thickness skin defect on day 3 post-surgery shows a large and diffuse area of granulation tissue (GT) and proliferated keratin. Under GT, extended edema and inflammatory cells, mainly neutrophils, have been identified (Figure 8).

**Figure 9:**
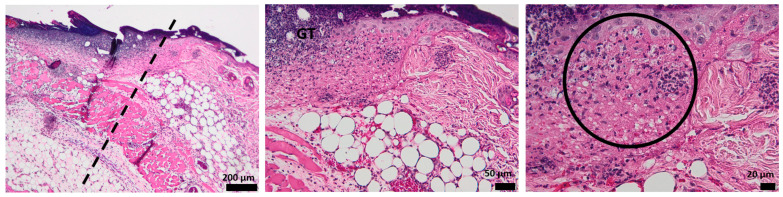
The histological aspect of the skin defect filled with HG at 3 days (H&E stain). Symbols: dotted line—separates healthy skin from the defect; GT—granulation tissue; circle—neutrophils.

The hydrogel-treated wound histology shows a reduction in the granulation tissue and less keratin on the surface. The edema area is reduced, and the presence of inflammatory cells is lower compared to the control (Figure 9).

**Figure 10:**
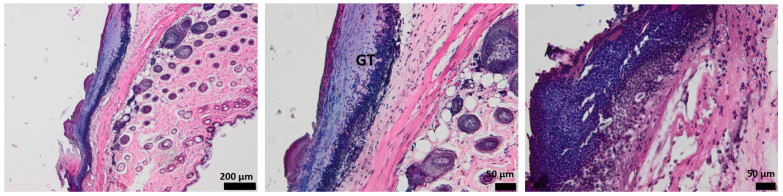
The histological aspect of the skin defect filled with HG_OMs at 3 days (H&E stain). Symbols: GT—granulation tissue.

When treated with oxygen-entrapping PLA microspheres embedded into a hydrogel dressing, an improved histopathological aspect of the skin is observed, with the collected micrographs showing a progressive healing process characterized by a good delimitation of the granulation tissue. Moreover, the edema is not present on the wound site, demonstrating the efficient activity of the dressing (Figure 10).

**Figure 11:**
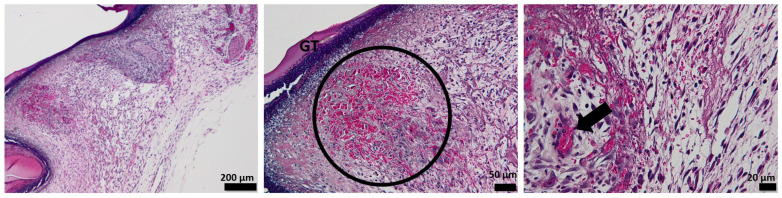
The histological aspect of the skin defect filled with HG-OMs_-MG at 3 days (H&E stain). Symbols: GT—granulation tissue; circle—extended vascular area; arrow—neutrophil extravasation.

Compared to the control and the previous wound dressings (HG and HG_OMs), the addition of Matrigel significantly improves the morphological aspect of the injured skin (Figure 11). The granulation tissue is well-defined and much thinner, in contrast to the explant results without Matrigel. Few inflammatory cells are observed, but sufficient to support the healing process.

The presence of Matrigel within the hydrogel dressing has a clear influence on the healing of skin tissue, with the angiogenesis process being activated and wound healing being well promoted.

In the first 3 days, regeneration processes that activate the acute inflammatory events are represented by neutrophils and granular tissue presence. In the case of Matrigel-enriched hydrogel, the process of forming new blood vessels is also observed.

### 3.4. Histological Aspect of the Wounds 7 Days after Injury (H&E Stain)

**Figure 12:**
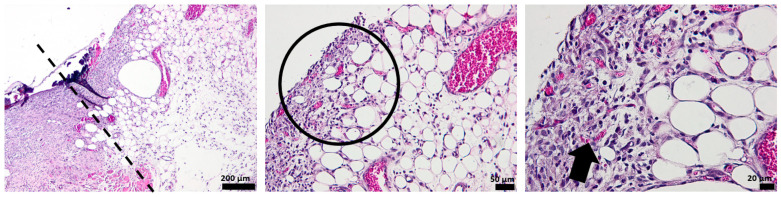
The histological aspect of the full-thickness skin defect at 7 days (H&E stain). Symbols: dotted line—separates healthy skin from the defect; circle, arrow—new capillaries.

The histological analysis of the un-dressing full thick skin 7 days after the surgery shows no epidermal formation. The area of the dermis formed by connective tissue is very weak, which is unusual for normal healing after 7 days, as it should form the skin. The arrow illustrates the vascularization that occurs after 7 days from the defect (Figure 12).

**Figure 13:**
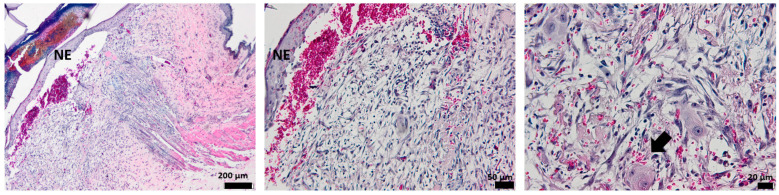
The histological aspect of the skin defect filled with HG at 7 days (H&E stain). Symbols: NE—new epidermis; arrow—epithelial cells in the dermis.

The tissue of the affected area treated with alginate hydrogel shows the formation of a new thin epidermis (NE), while the presence of isolated epithelial cells is also observed at the characteristic dermis area. The dermis shows a disturbed organization populated with collagen fibers, which makes the connection between the epidermis and dermis very fragile, with bleeding even occurring (Figure 13).

**Figure 14:**
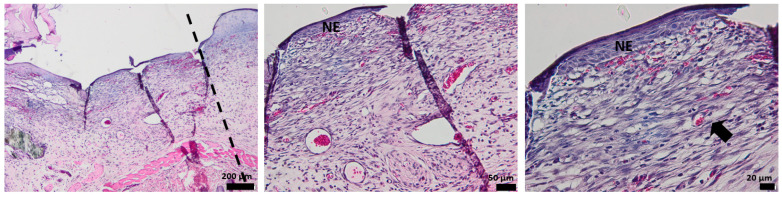
The histological aspect of the skin defect filled with HG-OMs at 7 days (H&E stain). Symbols: dotted line—separates healthy skin from the defect; NE—new epidermis; arrow—new capillaries.

After 7 days of treatment with the OMs-loaded hydrogel dressing, the formation of a new epidermis that tightly continues with a dermis can be noticed. Several collagen fibers are observed at the dermal level compared to the blank hydrogel, but the development of normal skin is not achieved. The presence of more capillaries, contrary to the control group, is evidenced by the arrow (Figure 14).

**Figure 15:**
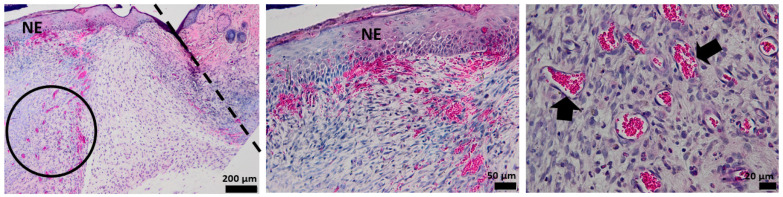
The histological aspect of the skin defect filled with HG-OMs-MG at 7 days (H&E stain). Symbols: dotted line—separates healthy skin from the defect; NE—new epidermis; circle—the proliferation of capillaries in a mature dermis; arrow—new capillaries.

The influence of Matrigel on the healing ability of the H_2_O_2_–PLA microspheres-loaded hydrogel dressing is evident after 7 days of defect occurrence (Figure 15). One can easily see the appearance of the new epidermis, which is thicker and better defined than in the case of HG-/HG_OMs-treated wounds. Its structure is characteristic of normal skin, so after 7 days, a normal epidermis is completely formed.

### 3.5. Histological Aspect of the Keratinization Process (Dane Stain)

**Figure 16:**
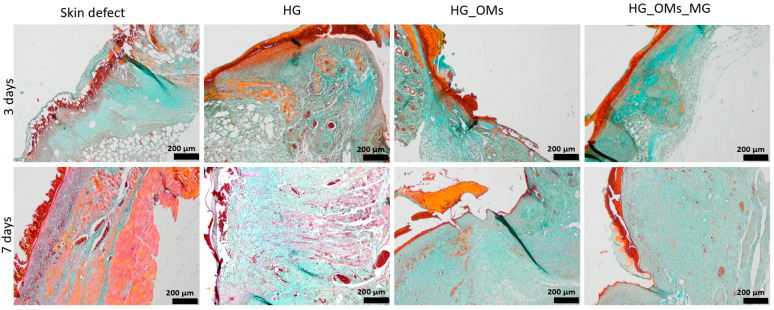
The histological aspect of the keratinization process during the healing process of the epidermis (Dane stain).

Dane coloring with orange/red-orange highlights the formation of keratin and pre-keratin, whereas the green color highlights the collagen in the skin. The collected micrographs of as-stained tissue specimens are included in Figure 16.

Three days after the surgery, all wounds begin a keratinization process. However, there is a notable difference in keratinization in uncovered skin defects compared to the skin treated with synthesized hydrogels. The difference is in defining keratinization more precisely in the epidermis, so the healing process is more advanced than untreated defects.

Moreover, in the case of the explant treated with the Matrigel-enriched hydrogel, it is noteworthy that collagen production (stained in green) is more intense than in the other experimental groups.

At 7 days, the histopathological aspects highlight the successful keratinization process. Cells that produce keratin and pre-keratin appear deeply in the epidermis and find themselves even in the papillary dermis, which is not shown in the other groups.

In the case of HG_OMs_MG-treated wounds, keratin is observed only in the epidermis, which correlates with the normal appearance of mature skin. In addition, the keratin layer is thinner and better defined.

These effects demonstrate similar characteristics with normal skin, so even after 7 days, the same HG_OMs_MG hydrogel is proven the most effective in treating wounds.

The collagen distribution differs depending on the material used, so after 7 days of the defect, collagen exhibits a normal appearance only for HG_Oms and HG_Oms_MG explants.

## 4. Discussion

Hydrogels have been extensively used in recent studies as promising materials for wound dressings suitable for different injuries [17], including diabetic foot ulcers. Polymers such as chitosan [65,66,67,68], alginate [65,68,69], gelatin [69], bacterial cellulose [70], and polyvinyl alcohol [66,67,71] have been reported as useful substrate materials for healing cutaneous lesions.

Given the attractive properties of polymeric materials in general and alginate in particular, this study explored the use of alginate-based hydrogels to enhance the healing of wounds in diabetic mice. The metabolic imbalance occurring in diabetic patients, in conjunction with diabetes-associated vascular conditions, causes a delayed and impaired healing process in skin injuries, generally resulting in chronic and complicated wounds [72,73]. As they undergo hypoxia, which alters the healing process through immune and cellular events, the fabrication of oxygen-releasing platforms emerges as a promising strategy for the local management of diabetic wounds [74,75]. Therefore, to improve the restorative potential of the herein-proposed hydrogels, oxygen-entrapping PLA microspheres were fabricated and incorporated into the base materials.

Following their sequential investigation, FT-IR analysis demonstrated the efficient fabrication of composite formulations (Figure 2). For instance, the signature ester of PLA was identified at ~1680 cm^−1^, a slightly shifted position when compared to other PLA-based formulations [76,77,78]. This was particularly assigned to the vibrations of the hydrogen-bounded carbonyl, which resulted in entrapping the H_2_O_2_ within the polymer matrix. Consistent with this observation, OMs had the most intense band in the hydroxyl region due to their dual origin (PVA and hydrogen peroxide). The formation of oxygen-enriched PLA-based microspheres was supported by the infrared data of HG_OMs hydrogels, which evidenced a decrease in the hydroxyl band but still a more intense signal compared to the pristine HG sample. In the case of HG_OMs hydrogels, cumulative overlapped signals for moieties in the 1000–1650 cm^−1^ region were evidenced, confirming their composite formulation. Moreover, the embedding of OMs within the hydrogel matrix was evidenced by the modifications that occurred in C-H vibrations, which switched from a sharper double-humped aspect (due to intense symmetric and asymmetric vibrations of PLA-originating methyl side groups) [79,80] to a reduced-in-intensity broader band (due to predominant vibrational modes of aliphatic C–H from the hydrogel matrix) [81,82]. Hydrogel formation was confirmed for HG and HG_OMs samples through the presence of alginate-originating asymmetric vibrations of COO– (~1600 cm^−1^) involved in ionic bonding [83,84].

PLA-based spheres have been extensively investigated as bioactive, non-immunogenic, and biodegradable carriers for controlled and targeted therapeutic formulations [85,86]. PVA addition is usually preferred to properly tune the hydrophobic nature, stability, and degradation rate of PLA-based systems, but also the loading efficiency of the therapeutic payload [87,88]. Using an adapted microemulsion protocol, hydrogen peroxide was easily and successfully embedded within polyester formulations. The H_2_O_2_-loaded PLA-based systems (OMs) displayed smooth surfaces, a spherical morphology, and micrometric size, with diameters ranging from 1 to 3 μm, as evidenced through SEM micrographs (Figure 3). Moreover, when added to the alginate hydrogel, the spheres maintained their dimensions and were evenly distributed both on the surface and within the hydrogel matrix (Figure 5).

The size order of the H_2_O_2_-PLA microspheres is consistent with other findings on the fabrication of oxygen-generating polymeric particles, with our spheres reaching even smaller dimensions than previously described. For instance, Nejati et al. [89] obtained PLA microparticles loaded with polyvinylpyrrolidone/hydrogen peroxide with average diameters between ~20 μm and ~90 μm. In comparison, Zhang and colleagues [90] synthesized oxygen-releasing polycaprolactone/calcium peroxide microspheres with sizes ranging from ~6 μm to ~30 μm.

Porosity represents an essential aspect that must be addressed when designing and fabricating functional wound dressings. The intrinsic porosity of polymer-based hydrogels is beneficial for providing biomechanical support, nutrient transport, and moisture retention while mimicking the natural local microenvironment and promoting cell ingrowth and migration, and later reparative/regenerative events, finally leading to structural and functional restoration [91,92]. The alginate hydrogels developed herein exhibited a favorable interconnected porous structure, with pores in the micrometric range (average pore size of 79.72 ± 2.12 and 110.25 ± 6.15 µm for HG and HG_OMs, respectively) (Figure 4 and Figure 5). These findings are in accordance with similar studies [93,94,95], as previously reported structures presented comparable architecture and pore sizes (i.e., 50–150 μm and 5–15 μm, respectively) [94,95]. SEM analysis revealed the interconnected and uniform macroporosity of HG hydrogel, but also the smooth surface of the pore walls. By contrast, the HG_OMs hydrogel exhibited increased heterogeneous porosity, with H_2_O_2_-PLA microspheres being distributed both onto and within the hydrogel.

Assessing the swelling behavior of dressing materials under biomimicking fluids provides essential information on their moisture absorption capacity, which is a relevant aspect for predicting their ability to absorb local exudate and edema. As SBF has similar ionic concentrations to human blood plasma, it was used in our study to evaluate the moisture uptake in hydrogel dressings (Figure 6a). It has been evidenced that both formulations had an important capacity to retain the fluid, starting from the first minutes of interaction and following a similar pattern. However, the addition of OMs determined a delayed dissolution of the hydrogel’s network, further resulting in increasing the SBF retention. This observation was better evidenced after 8 h of immersion due to the cumulative roles of OMs blocking the fluid from reaching the HG network and larger pores within the HG_OMs sample. Both hydrogels reached their maximal uptake capacity after 24 h, with ~160% and ~200% swelling rates being observed for HG and HG_OMs, respectively. After this time point, a decrease in their capacity to absorb the liquid occurred, finally resulting in ~140% (HG) and ~160% (HG_OMs) swelling rates. This decrease resulted from the degradation of both formulations (Figure 6b). A time-dependent loss in their mass was evidenced for HG and HG_OMs hydrogels, with no significant differences regarding the mass loss variation for samples of the same type. Though their porosity and fluid uptake rate were higher for HG_OMs, a reduced degradation was noticed for this hydrogel after the considered time points. This particular outcome was associated with the presence of PLA-based microspheres, which increased the stability of HG. Taking all into account, HG_OMs hydrogels showed increased interconnected microporosity, enhanced fluid retention capacity, and reduced degradation rate, being suitable for wound dressing applications.

The amount of H_2_O_2_, quantified using FT-ICR MS, was estimated as 1.97 ppm. During the inflammation of the tissue, the concentration of the H_2_O_2_ is predicted to reach millimolar levels. H_2_O_2_ levels are an important aspect of cellular functions and proliferation, but they can also cause cell death, which is necessary to generate at reduced levels in wounds [96,97].

In diabetic wounds, the impaired inflammation and proliferation phases of the healing process, local hypoxia, and inefficient immune and cellular responses lead to microenvironment-induced chronic hypoxia. Exacerbated and prolonged hypoxic conditions drastically intercept the angiogenesis process, further causing delayed and complicated healing [98,99]. In this context, wound oxygenation by means of topical oxygen therapy represents an emerging strategy for the healing of diabetic wounds, with promising therapeutic outcomes being reported for oxygen-releasing/-generating platforms [100,101] and oxygen-delivery/antioxidant formulations [102,103]. Herein, oxygen-releasing hydrogel dressings were proposed to promote angiogenesis for faster and more effective wound healing.

Our study evaluated the healing ability of hydrogel dressings on full-thickness dermal wounds of streptozotocin-induced diabetic mice. Following a 3-day and 7-day treatment, histopathological analysis was performed to collect information on the healing process. In comparison with untreated wounds, a reduction in local edema and inflammatory cells (3 days), but also the formation of the new epidermis and the presence of more blood vessels (7 days) were noticed after the treatment with HG dressings. These results are consistent with previous studies, validating alginate-based hydrogel’s high moisture absorption and pro-angiogenic effects [104,105]. Wounds treated with hydrogels embedding H_2_O_2_-PLA microspheres (HG_OMs group) showed no edema and significantly reduced inflammation after 3 days, while the formation of more and uniformly distributed capillaries and the presence of collagen fibers were evidenced after the longer treatment. As these findings were superior to the case of HG-treated wounds, we concluded that the oxygen released from polymeric spheres encouraged such beneficial outcomes, given the importance of oxygen in stimulating collagen production [106]. For what concerns the wounds treated with the HG_OMs_MG hydrogel dressing and in addition to the previous treatment group, well-defined granulation tissue and new capillaries were noticed after 3 days due to the structural support and biomolecule input provided by Matrigel [107] respectively. Moreover, a completely normal epidermis layer was observed, and a denser collagen network was observed after 7 days. Complementarily, a successful keratinization process occurred only in the epidermis of the HG_OMs_MG-treated wounds, representing a clear sign of mature skin.

The oxygen-releasing ability of the hydrogels incorporating PLA microspheres improved the skin’s histopathological aspect, allowed a progressive healing process, and supported angiogenesis. Even though both oxygen-enriched hydrogel formulations led to better wound healing results than the untreated skin defect and the pristine alginate-treated wound, the best outcomes were noted for the Matrigel-containing hydrogel dressing. These results were predictable, as Matrigel has been successfully employed in the design of wound dressings for skin repair and regeneration, owing to the synergistic action of constituent macromolecules and growth factors [43].

Our findings revealed that the HG_OMs_MG formulation offers the best healing outcomes, being able to almost close the skin defect 7 days post-injury. In comparison with other tested hydrogels, the HG_OMs_MG showed remarkable results, counting the lack of edema, erythema, or other clinical signs of inflammation, the presence of much thinner and better-defined granulation tissue, the normal aspect of mature skin, and the appearance of a new thicker and well-defined epidermis. In addition, the HG_OMs_MG dressings proved, stimulated (3 days), and promoted the angiogenesis process, as evidenced at 7 days post-injury by the abundance of newly formed capillaries. This result validated the faster wound healing ability of HG_OMs_MG hydrogel, as angiogenesis is known to play an important role during the healing of injured skin.

Thus, the developed hydrogel holds great promise in wound healing applications, being a desirable candidate for the proper treatment of skin defects in diabetic patients.

## 5. Conclusions

This study aimed to prepare a new oxygen-releasing dressing to promote angiogenesis for faster and more effective wound healing. The goal was achieved by embedding hydrogen-peroxide-containing PLA microspheres in an alginate-based hydrogel matrix (HG_OMs). Moreover, the addition of Matrigel in the hydrogel dressing (HG_OMs_MG) improved the therapeutic outcomes, as revealed by in vivo tests.

FT-IR analysis confirmed the formation of composite hydrogels, while SEM characterization evidenced their advantageous architecture. Specifically, H_2_O_2_-PLA microspheres were evenly distributed onto the surface and within the HG matrix, resulting in the formation of HG_OMs hydrogel, which exhibited a heterogenous interconnected porosity. This particular morphology is fitting for oxygen diffusion while allowing cell interaction and adhesion.

The suitability of the composite dressings in wound healing applications was confirmed by in vivo tests on diabetic mice. After 3 days from a skin injury, the wound healing starts with granulation tissue and acute inflammation progression, mainly characterized by neutrophil extravasation. The healing process was observed to be more advanced in the order HG_OMsMG > HG_OMs > HG > empty defect. After 7 days from a skin injury, the new epidermis was formed in all experimental groups, except the empty defect. The healing process (which includes the epidermis and dermis) and angiogenesis were effective in the order HG_OMs_MG > HG_OMs > HG.

In conclusion, by providing oxygen, extracellular matrix proteins, and specific growth factors, the HG_OMs_MG dressing could support diabetic wound healing in 7 days, holding promise as an alternative treatment strategy for diabetic foot ulceration.

## Data Availability

Not applicable.
